# Immune system and bone microenvironment: rationale for targeted cancer therapies

**DOI:** 10.18632/oncotarget.27439

**Published:** 2020-01-28

**Authors:** Antonio Gnoni, Oronzo Brunetti, Vito Longo, Angela Calabrese, Antonel-la Argentiero, Roberto Calbi, Giovanni Solimando Antonio, Antonella Licchetta

**Affiliations:** ^1^Medical Oncology Unit, “S. Cuore di Gesù” Hospital, Gallipoli, Italy; ^2^Medical Oncology Unit, National Cancer Research Centre, IRCCS IstitutoTumori “Giovanni Paolo II”, Bari, Italy; ^3^Radiology Unit, National Cancer Research Centre, IRCCS Istituto Tumori “Giovanni Paolo II”, Bari, Italy; ^4^Medical ThoracicOncology Unit, IRCCS IstitutoTumori “Giovanni Paolo II”, Viale Orazio Flacco, Bari, Italy; ^5^Radiology Unit, Ente Ecclesiastico “F. Miulli” Hospital, Acquaviva delle Fonti, Italy; ^6^Department of Biomedical Sciences and Human Oncology, Section of Internal Medicine “G. Baccelli”, University of Bari Medical School, Bari, Italy

**Keywords:** antiandrogens, bisphosphonates, bone niche, immune system, osteoimmunology

## Abstract

Osteoimmunology was coined about twenty years ago to identify a strict cross talk between bone niche and immune system both in physiological and pathological activities, including cancer. Several molecules are involved in the complex interaction between bone niche, immune and cancer cells. The Receptor Activator of NF-kB (RANK)/RANK Ligand (RANKL/Osteoprotegerin (OPG) pathway plays a crucial role in bone cells/cancer interactions with subsequently immune system control failure, bone destruction, inhibition of effect and metastasis outcome. The bidirectional cross talk between bone and immune system could became a potential target for anticancer drugs. Several studies evidenced a direct anticancer role with improved survival of bone-targeted therapies such as bisphosphonates and RANKL antagonist Denosumab. Conversely, initial data evidenced a possible anti-bone resorption effect of systemic anticancer drugs through and immunomodulation activity, i.e. new generation antiandrogens (Abiraterone) in prostate cancer. All data could open a future rationale of combined bone, immunologic and targeted therapies in cancer treatment.

## BIDIRECTIONAL CROSSTALK BETWEEN IMMUNE SYSTEM AND BONE NICHE: “OSTEOIMMUNOLOGY” CONCEPT

The “Osteoimmunology” concept was first evaluated in 2000 to identify a new interdisciplinary field, involving bone and immune system cells both in physiological and pathological activities [1]. The real news is to consider bone niche as a dynamic and complex system: all cells involved in the process interact with each other to continuous cycles of remodeling during human growth, with consequent and adequate bone growth [2].

Several recent data confirmed that bone cells works not alone in the processes of maintenance and accrual of bone mass. Specifically, also immune system play a crucial role in bone pathophysiology: several immune cells and immune-related factors, such as Interleukins (i.e., IL-6, -11), Tumor Necrosis Factor (TNF)-a, Nuclear Factor of Activated T-cell, cytoplasmatic-1 (NFATc1) [3–9] interact with bone cells to the bone “equilibrium”. Surprisely, recent data demonstrated a bidirectional cross talk between immune system and bone cells, assuming a feedback mechanism. For example, Zhu and Miller in their works showed a direct activity of osteoblasts to of B-lymphocytes differentiation from hematopoietic stem cells [10], by an osteoblasts secretion of Interleukin (IL)-7 and C-X-C motif chemokine Ligand (CXCL)12 [11]. On the other hand, many cytokines as IL-1, IL-15 and IL-17f increase osteoblast activity [7]. In addition, osteoclasts regulate immune cells activity also indirectly through osteoblasts, by the secretion of cathepsin K and the T Cell, Immune Regulator 1, ATPase, H+ transporting, lysosomal V0 protein A3 (Tcirg1) [12, 13].

In addition, several other immune cells and factors promote osteoclastogenesis, such as: neutrophils, synoviocytes, T-cells, Activated leucocytes, dendritic cells, stimulated stromal cells, Macrophage Colony Stimulating Factor (M-CSF), Natural Killer (NK)-cells; IL-1a, IL-1b, IL-7, IL-17, IL-15, IL-8, TNFa, IL-23, IL-24, IL-34 [14–22]. The bone niche with immune cells is a real “place of call” for tumor and cancer stem cells (CSCs) [23]. We can find in bone marrow different immune cells, such as granulocytes, macrophages, dendritic cells (DC), NK cells, T and B lymphocyte subsets, and myeloid derived suppressor cells (MDSCs). In spite of their great number and variability in bone niche, immune cells appear not able to control the processes of cancer cells growth and metastatization [23]. Probably, it is due to the presence in bone niche of contextual immature and suppressor immune cell types, such as T regulatory cells and MDSCs. Specifically, Feuerer et al. demonstrate that infiltrating T regulatory cells produce RANKL, with immune system downregulation and osteoclast differentiation. This process lead to osteoclastogenesis and bone metastasis [24]. Moreover, NK cells showed an ambiguous role in bone niche. In several experiments in melanoma, prostate, and breast cancers, they present an antitumor activity [25]. On the other hand, several data showed that NK cells promote melanoma cells proliferation and CSC phenotype conversion into bone niche. Furthermore, B and T cells contribute to the process of osteoclastogenesis, by the production of different factors such as TNFα and RANKL [26].

The turning point was in 1990, when the Receptor Activator of NF-kB (RANK)/RANK Ligand (RANKL)/Osteoprotegerin (OPG) system was discovered with its critical role in regulating osteoclastogenesis and bone remodeling activity [27]. While the RANK receptor is present on the surface of mature osteoclasts, RANKL is produced in a soluble form by osteoblasts, stromal cells and immune cells. The soluble receptor OPG plays an antagonist role against RANK/RANKL interaction, blocking the activity and the maturation of osteoclast [28, 29]. The equilibrium between RANK and OPG is regulated by activity of several cytokines and systems, as interleukin (IL)-1, IL-6, TNF alpha, TNF receptor-associated factors (TRAFs), PI3K, c-Src, Akt/PKB and mTOR [30, 31]. Several data evidenced that many of these factors are also involved in immune system regulation. Moreover, RANK/OPG balance plays a fundamental role in immune system activity: it increases lymphocyte development in lymph nodes, sustains the activation and the maturation of DC, and regulates the immune response mediated by T cells [32, 33].

Furthermore, pathological conditions show this close interaction between bone and immune system. Initial data evidenced that several bone diseases present an immunologic origin, such as rheumatoid arthritis, osteoarthritis and osteoporosis. There is a rise in the existence of these different skeletal diseases, which occur because of defective bone remodeling as a consequence of skewed immune system because of disruption of the homeostatic nexus between immune system and bone cells, that enhanced bone loss [34].

In 2014, Krevvata et al. evidenced a correlation between bone niche and cancer cells in acute myeloid leukemia (AML). In this case, osteoblasts promote the progression and transformation of the myeloid cells lineage in preneoplastic and neoplastic cells. Specifically, authors demonstrated that osteoblasts are able to slow down leukemia progression through an unfavorable microenvironment for leukemic blast growth. The “bone niche” concept becomes a “niche-induced leukemia” system: for the first time bone niche is evaluated as a dynamic system that include bone, immune and cancer cells [35].

## ALTERATIONS OF BONE AND IMMUNE SYSTEM IN CANCER: PRECLINICAL DATA

Several preclinical data in the last years demonstrated that cells involved in bone microenvironment and immune system can promote tumor growth and progression. Bone represents a cancer cells sanctuary against anticancer therapies. Many authors suggested that the bone niche probably guarantees an evasion of the immune system by disseminated tumor cells. Furthermore, bone niche preserve cancer cells from anticancer drugs [36]. The process of hematopoiesis occurs in skeleton and is guaranteed by the bone niche, in which different cytokines, growth factors and adhesion molecules play a crucial role [37]. The same bone niche, however, with the involvement of the near microenvironment, became a “soil” for the development of several tumor cells, including primitive hematological cancers and metastatic solid tumors [38].

**Figure 1:**
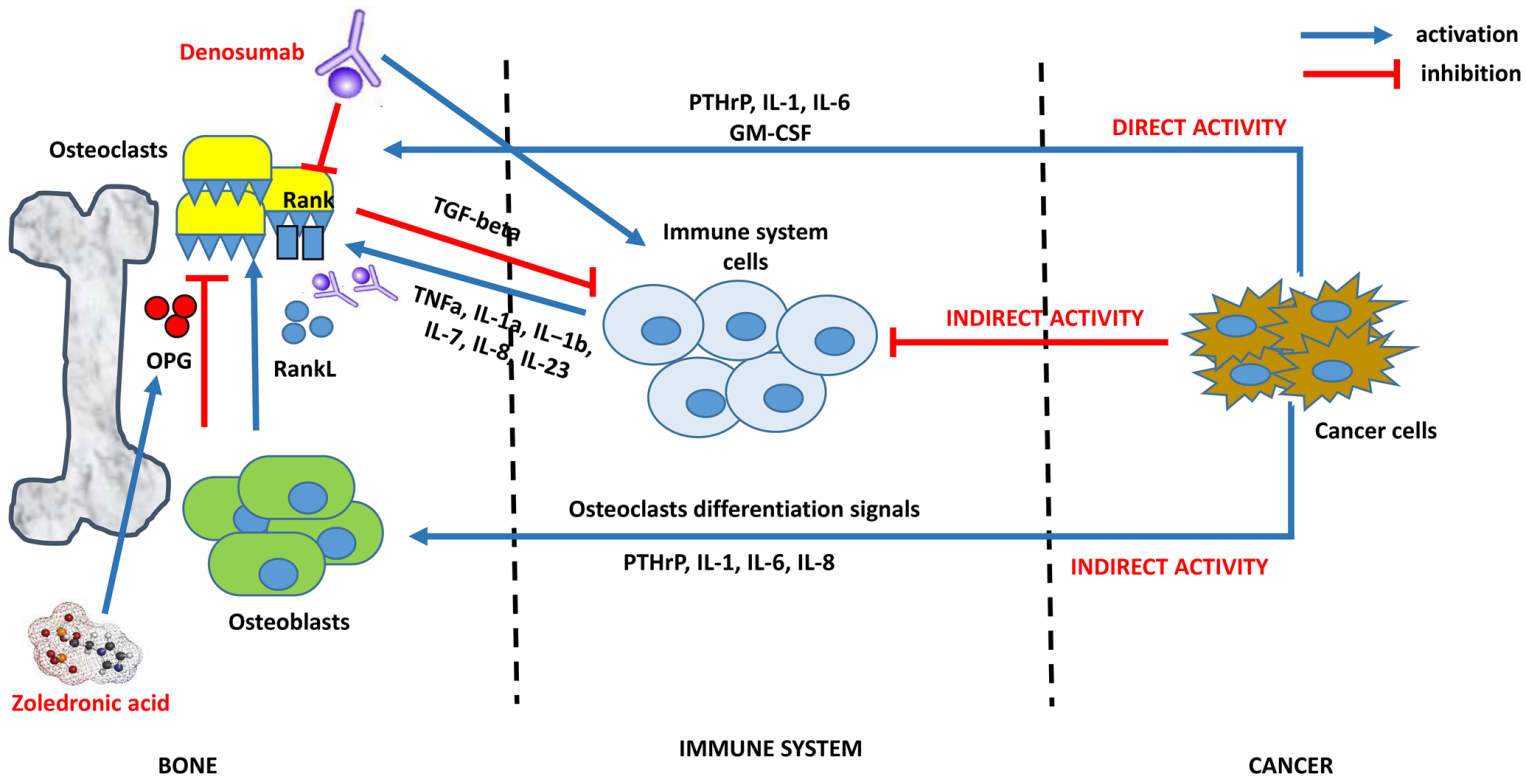
An example of interaction between bone, immune and cancer cells: osteoclastogenesis, mechanism of bone resorption and potential targets of biphosphonates and denosumab. The complex process of osteoclastogenesis in cancer is regulated by an interaction between bone, immune and cancer cells. Cancer cells promote this process in two ways: 1) indirectly, stimulating osteoblast to activate the RankL/Rank pathway (bone osteoclastogenesis) and deregulating immune cells activity against osteoclasts; 2) directly, stimulating osteoclastsogenesis by upregulation of IL-1, IL-6, PTHrP, GM-CSF. After cancer cells signals, immune system cells activate osteoclastogenesis by upregulation of TNFa, IL-1a, IL-1b, IL-7, IL-8, IL-23. Once activated, osteoclasts protect their growth with the inactivation of immune system by TGF-beta production. Bisphosphonates (i.e. Zoledronic Acid) inhibit osteoclast formation, recruitment and adhesion to bone shift the balance towards OPG production by osteoblasts and induce osteoclasts apoptosis. Denosumab is a fully human monoclonal antibody with anti-RANKL activity, thus inhibiting osteoclast activation by Rank receptor. Initial data evidenced a possible role in immune system preservation by B cell/T cell differentiation and dendritic cell survival.

In addition, tumor cells are able indirectly to reduce their immunogenicity by bypassing tumor immune surveillance mechanism. Although we know little about the immune system remodeling by bone homeostasis, some possible mechanisms begin to be demonstrated. In the bone niche, cancer cells are able to overbalance the RANKL/OPG ratio to osteoclastogenesis, favoring bone resorption and metastases implant. The osteoclastogenesis process leads bone niche to down regulation of immune system pathway, in a vicious circle that enhances tumor bone spread. Several works demonstrated these processes: i) RANK-expressing tumor cells/RANKL activation determines tumor metastatization; ii) T-cell suppression in bone-tumor niche helps bone lysis and tumor cells implantation; iii) T-cell suppression reduces osteoblastogenesis and bone stabilization; iv) Osteoclast factors enhance tumor spread through the inhibition of the T cells proliferation; v) Bone niche immune cells, as macrophages, can participate in antitumor responses after anticancer therapy, with elimination of circulating tumor cells and reduction of bone cancer cells implantation; vi) RANKL/RANK/OPG pathway actually represents a negative prognostic factor in cancer development [39–43] (Figure 1).

Prostate cancer is one of tumors that has been analyzed more frequently as regards bone, cancer and immune cell activity. Tumor cells increase the RANKL/OPG ratio indirectly in the bone with the release of different factors such as PTHrP, IL-1, IL-6 (osteoclast differentiation and survival activity) and directly with osteoclast precursors interaction and co-activation. In addition, cancer cells produce osteoblast differentiation inhibitors such as dickkopf-1 (DKK-1) and activin A [44, 45].

Recently, other molecules, such as PGE2, have been identified as possible “actors” in the processes of bone resorption and cancer metastatization, especially in patients with prostate cancer. Probably, tumor cells wedged in bone niche deregulates bone remodeling and manifests as osteolytic lesions that may cause skeletal related events (SREs). According these data, PGE2 could became a possible future therapeutic target in the treatment of prostate cancer [45].

In addition, multiple myeloma (MM) is a hematologic malignance that depend from the clonal expansion of malignant plasma cells within the bone marrow. This disease is often associated with adverse SREs. The bone lesions in MM are always lytic and it depends their ability to promote the processes of bone resorption. The principal mechanism is the displacement of the RANKL/OPG ratio to the process of osteoclastogenesis [46]. For example, Schramek et al. recently demonstrated that aberrant RANK/RANKL signaling in mammalian tissues promote bone resorption and the rapid development of progestin-mediated breast cancer. This process is amplified by a synergistic immune cells deregulation, mediated by cytokines such as TNF-alpha and IL-6 [47]. The aberrant activity RANK/RANKL pathway promotes the bone niche invasion by the RANK-expressing mammary epithelial cells. Moreover, the RANK/RANKL upregulation promotes antiapoptotic processes in response to DNA damage. The blockade of RANK/RANKL signaling in mice experiments (using genetic ablation of Rank or RANKL-Fc) inhibits the development of mammary tumors [48].

For this reason, RANK/RANKL/OPG system became a future therapeutic target, with possible prevention of bone resorption and decreasing of the number of SREs. Interestingly, direct antitumor effect through reactivating immune system cells. For the first time we can consider a bone target therapy as a possible systemic anticancer effect.

## BONE TARGETED DRUGS HAVE A ROLE IN ANTICANCER SYSTEMIC THERAPY THROUGH IMMUNE SYSTEM ACTIVATION

Bisphosphonates were the first drug class that demonstrates the ability to inhibit osteoclast formation, recruitment and adhesion to bone shift the balance towards OPG production by osteoblasts and induce osteoclasts apoptosis (Table 1). The first effect was to prevent pathological bone resorption with a dramatic SREs reduction [49].

**Table 1:** Bisphosphonates (zoledronic acid) and denosumab in patients with bone metastases: current demonstrated efficacy in different cancer types

Zoledronic acid	Multiple myeloma [48, 49]
	Breast cancer [48, 49, 50]
	Lung cancer [64]
	Renal cell carcinoma [53]
	Prostate cancer [49]
Denosumab	Multiple myeloma [58]
	Breast cancer [59, 60]
	Prostate cancer [59, 60, 62, 63]
	Hepatocellular carcinoma [55]
	Ewing Sarcoma [65]

Clezardin et al. in a recent study also demonstrated the existence of direct and indirect antitumor activity. Bisphosphonates counteract the tumor proliferation in bone niche reducing the release of bone-derived growth factors and cytokines. In addition, other mechanism could be the inhibition of tumor cell adhesion and invasion, and the apoptosis of cancer cells (bidirectional bone/cancer cells interaction) [50]. The latest data regarding third generation bisphosphonates evidenced also an indirect antitumor effect via immune system regulation. zoledronic acid and pamidronate activate T cells surveillance in bone niche and blood, with antiangiogenic and immune-modulatory mechanism [51]. Fournier et al. showed that zoledronic acid at therapeutic dose promotes the activity of T cells and the blockade of osteoclast-mediated bone resorption (“dual inhibition”) [52].

Breast cancer, MM and prostate cancer are the tumors with zoledronic acid activity which have been studied more [53]. Several data confirmed that zoledronic acid in advanced breast cancer patients prevents the bone loss induced by aromatase inhibitor use, bone metastases, SREs and reduced survival [54]. Recent data also evidenced a possible synergistic effect of zoledronic acid with chemotherapy (such as cisplatin) in metastatic triple negative breast cancer, with results in the prolongation of progression free survival (PFS) and overall survival (OS). Specifically, zoledronic acid stimulates the number of T cells and monocytes, and inhibits the process of osteoclast-mediated bone resorption (bone/cancer/immune cells interaction) [55]. Recent data suggest that bisphosphonates could exert a protective activity to bone reducing the bone niche invasion and metastatization by cancer cells. It represent a clear anticancer activity in postmenopausal breast cancer women treated with adjuvant hormonal therapy, hypothesizing that an early use in adjuvant setting could provide the greatest benefits [56]. In addition, in renal cell cancer, lung cancer and hepatocellular carcinoma recent data evidence a systemic activity of bisphosphonates to prolong patient survival and increase quality of life [57–59, 64].

Recently, a new drug blocking RANK/RANKL/OPG pathway exerts bone control with the prevention of bone resorption and destruction: denosumab, a fully human monoclonal antibody (anti-RANKL), authorized for the treatment/prevention of SREs in bone metastases from MM, breast, prostate cancer and Ewing Sarcoma. Better than zoledronic acid, denosumab decreases the number of SREs, delays SREs onset, reduces bone pain and prevents immune system preservation by the differentiation and survival of B cell, T cell and DCs (both in bone niche and blood) [60, 65]. Initial preclinical data evidenced a possible role in systemic tumor control with better progression and overall survival, but strongly results are warranted. In addition, in this case the systemic anti-cancer effect seems due to a better immune system regulation, T-cell activation and immunogenic chemokine’s increase [61]. Current clinical studies are evaluating to a greater extent the effect of denosumab on survival and other biomarkers.

## ANTICANCER DRUGS PLAY EFFECT AGAINST BONE DISRUPTION THROUGH IMMUNOMODULATION: FIRST SMALL STEPS

On the other hand, considering the bone/cancer cells interaction as a bidirectional process, some authors first evaluated a possible converse scenario: a bone disease control using the systemic anticancer drugs. Certainly, a better control of systemic cancer disease delays metastases onset, including bone. Recently authors evidenced an indirect bone disease control by new generation antiandrogens, such as the CYP17 inhibitor Abiraterone in prostate cancer [62]. In all clinical studies, Abiraterone demonstrates a better control of systemic disease also thanks to bone resorption control (prolonged radiographic progression free survival), SREs reduction (time to first SRE), better quality of life with reduced bone pain. Laboratory data evidenced a possible bone niche control by immune system activation, such as T-cells increase, DCs control and immune-stimulatory cytokines. Detti et al. recently also evidenced a possible synergistic activity of abiraterone with radiotherapy in bone metastases control in patients with advanced prostate cancer. Radiotherapy in bone niche activates immune system control and exposes immune cells to abiraterone activity. The result is the block of the bone/cancer cells pathway, with osteoclastogenesis reduction and bone stabilization [63]. We are only at the beginning of this new aspect of osteoimmunology and further data are necessary to better clarify this bone disease control through immunomodulation.

## CONCLUSIONS

Osteoimmunology is a new field in the last years, with a great relevance to the control of bone homeostasis. Its discovery has changed the therapeutic scenario in cancer bone disease. Until the 2000s, bone niche has been evaluated as an “impenetrable sanctuary” for anti-cancer drugs. Its infiltration by cancer cells has represented a defeat for oncologic treatments, an early progression signal with poor prognosis. After a correct knowledge of the dynamic system of bone niche and bone/immune cells pathways, the osteoimmunology concept has allowed to develop different potential mechanisms involved in pathologic operation of bone remodeling system. Actually, the effects on bone of several immune cells (such as macrophages, granulocytes and innate immune pathways) remain unclear. A better understanding of the molecular interaction between the three actors of this dynamic system (bone, immune and cancer cells) is almost necessary. A bidirectional process between cancer cells and bone niche components could explain a possible both locoregional (bone) and systemic cancer control. We could hypothesize that the immune system represents a “bond”, a bridge between bone niche cells and cancer cells. An adequate knowledge of this complex equilibrium can represent a potential therapeutic target to control not only bone metastases, but also systemic cancer pathology. Moreover, after the recent advent of immunotherapy in anticancer drugs scenario, all data could open a future rationale of combined bone, immunologic and targeted therapies in cancer treatment.
